# Angiopoietin-2: Modulator of Vascular Permeability in Acute Lung Injury?

**DOI:** 10.1371/journal.pmed.0030113

**Published:** 2006-02-07

**Authors:** Tomoki Hashimoto, Jean-Francois Pittet

## Abstract

Hashimoto and Pittet discuss a new study in PLoS Medicine that suggests that angiopoietin-2 may have a key role to play in causing ARDS in patients with sepsis.

Tie (tyrosine kinase with immunoglobulin-like loop and epidermal growth factor homology domains) represents a novel class of receptor tyrosine kinases that are mostly expressed by vascular endothelial cells. There are currently two known members in this class: Tie1 (also described as Tie) and Tie2 (also known as Tek; reviewed in [ 1]). During embryonic development, endothelial cells express both Tie1 and Tie2 receptors. Tie2 is also expressed in quiescent endothelial cells in adult tissues. Unlike Tie1, Tie2 has well-described ligands called angiopoietins. Of the four currently known angiopoietins (Ang1–4), the best characterized are angiopoietin-1 (Ang1) and angiopoietin-2 (Ang2).

The functional consequences of Ang/Tie2 signaling have been well established through genetic loss-of-function and gain-of-function experiments in animals and cultured human endothelial cells. According to these functional studies, Ang1 is an agonist ligand that activates Tie2, thus controlling endothelial cell survival and vessel maturation associated with the quiescent nonproliferating endothelial cell phenotype. The functions of Ang2 appear more complex. Ang2 binds to the Tie2 receptor, but acts as a non-signal-transducing Tie2 antagonist ligand that blocks Ang1/Tie2 signaling and acts as a blood vessel–destabilizing cytokine. However, high concentrations of Ang2 or prolonged exposure of endothelial cells to Ang2 have been shown to activate Tie2 signaling, although the mechanisms of this paradoxical agonist activity of Ang2 are not well understood [ 1].

## The Role of Angiopoietins in Angiogenesis

Angiopoietins are critically involved in physiological and pathological (e.g., tumorigenesis) blood vessel formation, or angiogenesis (reviewed in [ 2]). Neither Ang1 nor Ang2 can trigger an angiogenic response alone, but both enhance vascular endothelial growth factor (VEGF)–induced angiogenesis. Ang1 signaling via Tie2 promotes vessel maturation and quiescence, whereas Ang2 blocks Ang1/Tie2 signaling, and this leads to either angiogenesis or vessel regression and apoptosis, depending on the presence of VEGF or other angiogenic factors.

The current working hypothesis is that new vessel formation requires the temporal and spatial integration of signals originating from both Tie2 and VEGF receptors. The first step is an upregulation of Ang2 that blocks the stabilizing action of Ang1 on the vasculature. This then allows VEGF and other growth factors to promote endothelial cell migration, proliferation, and organization into new vessels. Once the new vessels have been formed, Ang1 levels increase and Ang2 levels decrease, allowing the formation of a mature vasculature. This hypothesis suggests that the Ang1/ Ang2 ratio, more than absolute levels of either ligand, is critical for determining the endothelial cell phenotype [ 2].

## Angiopoietins and Inflammation

The expression and phosphorylation of the Tie2 receptor in the normally quiescent mature vasculature suggests that Ang1/Tie2 signaling not only plays a role in angiogenesis but is also actively involved in the formation and maintenance of the vascular endothelial barrier. Indeed, the binding of Ang1 to Tie2 stabilizes in vitro endothelial cell interactions with the extracellular matrix and junctional proteins, and enhances barrier function [ 3]. For example, Ang1 has been shown to protect the adult vasculature against plasma leakage induced by VEGF [ 4] by inhibiting the calcium influx into the cells [ 5]. In addition, the activation of Ang1/Tie2 signaling attenuates H2O2-induced apoptosis by activating the phosphoinositol 3-kinase/Akt pathway [ 6]. These results suggest that Ang1 has anti-inflammatory properties.

A recent study showed that the adenoviral gene transfer of Ang1 can protect mice against endotoxic shock induced by Escherichia coli endotoxin, and is associated with an improvement in hemodynamic function, reduced lung injury, and a lower expression of inflammatory adhesion molecules [ 7]. A similar protective function of Ang1 was also observed in mice that had increased brain barrier permeability due to overexpression of VEGF [ 8]. In contrast, Ang2 appears to cause vascular leakage. It stimulates in vivo within 30 minutes the extravasation of fluid in the mouse paw [ 9]. When submaximal doses of Ang2 and VEGF were administered at the same time in the mouse paw, their effects were additive, suggesting that the two factors act independently [ 9]. Furthermore, expression profiling studies have shown that endothelial cells are the primary source of Ang2 [ 10], and that Ang2 levels can be transcriptionally and post-transcriptionally regulated by hypoxia or exposure to growth factors, such as VEGF or platelet-derived growth factor (PDGF) [ 11]. In addition, Ang2 protein is stored inside endothelial cells and can be secreted within minutes after stimulation by thrombin or histamine [ 12]. These findings indicate that Ang2 is not only a regulator of angiogenesis and vessel maturation but is also involved in rapid vascular homeostatic reactions such as inflammation and coagulation.

## A New Study: The Role of Ang2 in Acute Lung Injury

In a new *PLoS Medicine* study, Samir Parikh and colleagues [ 13] describe a new, important role for Ang2 in acute lung injury. In a series of elegant and complementary human and mouse in vivo and in vitro studies, the investigators demonstrate that circulating Ang2 is elevated in humans with sepsis and impaired oxygenation. Furthermore, their study shows that the serum of these patients disrupts in vitro the endothelial architecture, an effect that correlates with the serum level of Ang2 and that can be reversed by the addition of Ang1. These results suggest that the presence of Ang2 in serum is at least in part responsible for compromising the barrier function of the vascular endothelium in acute lung injury from sepsis.

Additional in vitro experiments demonstrated that Ang2 alone can replicate the effect of human septic serum on endothelial structure, and that it promotes endothelial hyperpermeability via Rho-kinase and myosin light chain kinase activation. Similar effects were observed when Tie2 levels were reduced with Tie2 small interfering RNA. These results suggest that Tie2 signaling is constitutively active in endothelial cells and that the addition of Ang2 blocks Tie2 signaling by inhibiting Tie2 phosphorylation, leading in turn to Rho-kinase and myosin light chain kinase activation and disruption of the endothelial monolayer. A final set of in vivo studies demonstrated that systemic administration of Ang2 directly provokes vascular hyperpermeability and pulmonary edema in healthy adult mice.

## Unanswered Questions

Despite the importance of these results, several questions about the role of Ang2 in acute lung injury remain unanswered. First, as previously shown for angiogenesis, the Ang1/Ang2 ratio may be more important than the absolute Ang2 levels for determining the effect of Tie2 signaling on endothelial cell permeability. Parikh and colleagues' study supports this hypothesis because recombinant Ang1 was able to reverse the permeability effect of human septic serum containing high levels of Ang2 on quiescent human vascular endothelial cells.

Second, it remains unclear whether Ang2 only acts as an antagonist of the Tie2 signaling or whether it has agonist Tie2 effects on the pulmonary vasculature of patients with acute lung injury. Previous studies have reported that Tie2 mRNA and protein are highly expressed in the lungs [ 14], indicating a potential role for this pathway in controlling lung endothelial permeability. Furthermore, Ang2 has been shown to have Tie2 agonist properties in the presence of other angiogenic factors such as VEGF [ 10], and high plasma levels of VEGF have been reported in patients with sepsis and correlate with increased vascular permeability [ 15]. Increased VEGF gene and protein expression has also been associated with ischemia-reperfusion lung injury [ 16].

Third, another intriguing question is which organs or cell types are responsible for elevated serum Ang2 in patients with severe sepsis. Is elevated Ang2 a manifestation of a localized increase in Ang2 production in the lungs? Or is increased Ang2 production a universal response of the vascular system during sepsis since sepsis affects the entire vasculature indiscriminately? If the latter is the case, vascular leakiness caused by Ang2 in severe sepsis may not be unique to the lungs, but may be a systemic phenomenon that affects other vital organs, including the brain.

Fourth, the question, “Can soluble Ang2 serve as a biomarker of acute lung injury?” remains an open one. A clinically useful marker should have a high positive predictive value in patients who are at risk for the syndrome but have not yet developed symptoms. Furthermore, a suitable biomarker should also be highly discriminative in predicting mortality in these patients. Although, as acknowledged by the authors, a much larger clinical study would be required to definitively conclude that the serum level of Ang2 is a valid biomarker to predict subsequent acute lung injury, the clinical data collected by Parikh and colleagues are not very encouraging. In this small cohort of patients, serum Ang2 levels reach their peak only at the same time as the nadir of the PaO _2_/FiO _2 _ratio. Furthermore, there was no difference in serum levels of Ang2 between patients who died and patients who recovered.

## Conclusion

In summary, Parikh and colleagues have provided important new insights regarding the mechanisms that control lung vascular permeability in patients with acute lung injury. They have demonstrated that Ang2, one of the known ligands of the Tie2 receptor, has a significant role in increasing vascular permeability in patients with acute lung injury. As with all interesting discoveries, their study also raises many questions, and makes it clear that more work is needed to understand the complexity of the control of the lung vascular permeability in vivo under pathological conditions, such as acute lung injury.

## Abbreviations

Ang1: angiopoietin-1
Ang2: angiopoietin-2
VEGF: vascular endothelial growth factor
